# OUTBREAK: a user-friendly georeferencing online tool for disease surveillance

**DOI:** 10.1186/s40659-021-00343-5

**Published:** 2021-07-08

**Authors:** Raúl Arias-Carrasco, Jeevan Giddaluru, Lucas E. Cardozo, Felipe Martins, Vinicius Maracaja-Coutinho, Helder I. Nakaya

**Affiliations:** 1 Advanced Center for Chronic Diseases – ACCDiS, Facultad de Ciencias Químicas y Farmacéuticas, Universidad de Chile, Santos Dumont, 964, Independencia, 8380494 Santiago, Región Metropolitana Chile; 2 Department of Clinical and Toxicological Analyses, School of Pharmaceutical Sciences, University of São Paulo, Av. Prof. Lúcio Martins Rodrigues, 370, Block C, 4th Floor, São Paulo, SP CEP 05508-020 Brazil; 3 Instituto Vandique, João Pessoa, Brazil; 4 Scientific Platform Pasteur USP, São Paulo, Brazil; 5 Hospital Israelita Albert Einstein, São Paulo, Brazil; 6 Instituto Todos pela Saúde, São Paulo, Brazil

**Keywords:** Outbreak, Pandemic, Epidemiology, Surveillance, Georeferencing

## Abstract

The current COVID-19 pandemic has already claimed more than 3.7 million victims and it will cause more deaths in the coming months. Tools that track the number and locations of cases are critical for surveillance and help in making policy decisions for controlling the outbreak. However, the current surveillance web-based dashboards run on proprietary platforms, which are often expensive and require specific computational knowledge. We developed a user-friendly web tool, named OUTBREAK, that facilitates epidemic surveillance by showing in an animated graph the timeline and geolocations of cases of an outbreak. It permits even non-specialist users to input data most conveniently and track outbreaks in real-time. We applied our tool to visualize the SARS 2003, MERS, and COVID19 epidemics, and provided them as examples on the website. Through the zoom feature, it is also possible to visualize cases at city and even neighborhood levels. We made the tool freely available at https://outbreak.sysbio.tools/. OUTBREAK has the potential to guide and help health authorities to intervene and minimize the effects of outbreaks.

## Background

Effective epidemiological surveillance is essential to ensure a timely and adequate response to infectious disease outbreaks. Communicable disease surveillance provides requisite information to monitor, evaluate, and model the preventive and control measures. The primary goal of the process is to monitor the spread of an ongoing infectious disease outbreak and geographically detect disease hotspots. Moreover, it assists in tracking emerging diseases that pose a threat to public health across the globe. Integrated disease surveillance enables health authorities to (i) identify populations at risk, (ii) implement prevention and control strategies, (iii) detect unusual disease patterns, and (iv) contain the re-emergence or emergence of communicable diseases [1].

The coronavirus epidemics (SARS, MERS-CoV) and the COVID-19 pandemic have displayed the capacity of an infectious disease to spread rapidly. The SARS epidemic (2003–2004) infected over 8000 people in 17 countries with a mortality rate of 9.6% [2]. Later in 2012, MERS-CoV infected 2519 subjects with a mortality rate of 34.3% in 27 countries [3]. As of June 2021, the SARS-COV-2 pandemic has already claimed more than 3.7 million lives worldwide, and more deaths are projected in the coming months [4]. Coronaviruses spread through direct human contact and objects contaminated by respiratory droplets exhaled by the infected persons [5]. The high infection rate of SARS-COV-2 [6] caused a large proportion of the population to be infected and caused a country's health system to collapse, such as what happened to Italy in 2020 [7]. In such a scenario, tools that can track the numbers and pinpoint the location of cases become critical to implementing effective policies by the governments to control the spread of the disease [8, 9].

The current COVID-19 pandemic led several research teams to develop web-based dashboards that display surveillance datasets generated across the globe. Examples include COVID-19-Map (https://coronavirus.jhu.edu/map.html) by the Johns Hopkins University [10], COVID-19 Surveillance Dashboard (http://nssac.bii.virginia.edu/covid-19/dashboard/) by the University of Virginia, the World Health Organization’s (WHO) dashboard (https://who.maps.arcgis.com/apps/opsdashboard/index.html), and COVID-19 local dashboards from Bing-Microsoft (https://www.bing.com/covid) and Google (https://news.google.com/covid19/map). HealthMap (https://healthmap.org/en/), operated since 2006, developed by Harvard University and Boston Children’s Hospital, also tracks other infectious diseases such as influenza, dengue, tuberculosis, and measles. Although most of these dashboards use open-source data, they run on Esri ArcGIS web services which are often expensive or hard to build since it demands a specific knowledge of GIS-based software and programs. In addition, these dashboards are limited to monitor only from a spatial epidemiological perspective and usually lack a time axis to track the evolution of an outbreak. Moreover, these dashboards do not allow the user to input data, restricting non-GIS specialists to utilize the dashboard features for their research or decision-making, who usually lack access to such software or services. To overcome these issues, we developed a new web-based tool that allows the user to input epidemiological data in a user-friendly way to track, study and visualize the outbreaks in real-time.

## OUTBREAK Overview

OUTBREAK facilitates epidemic surveillance by visualizing user-defined geographical coordinates (geolocations of cases) on an interactive map and generating an animated timeline graph (case numbers). The tool is available for access through a website at https://outbreak.sysbio.tools/, where even non-specialists can input data in the most convenient way. It accommodates worldwide epidemic surveillance i.e. continent, country, regions, state, municipality, and street. For example, we generated visualizations showing the evolution of the 2003 SARS epidemic (https://outbreak.sysbio.tools/animation/SARS_2003), the ongoing COVID-19 pandemic with infected and death cases, and another with deaths and vaccines data from January 1, 2020, to May 31, 2021 (https://outbreak.sysbio.tools/animation/COVID19 and https://outbreak.sysbio.tools/animation/COVID19vax, respectively).

## Availability and codes

OUTBREAK online tool is freely available under MIT license at https://outbreak.sysbio.tools/. The software includes a text and video tutorial with a detailed description of how to use it. OUTBREAK uses Flask, a Python-based web framework on the server-side [11]. The user interface is built using JavaScript through the React.js (reactjs.org) library on a Node.js environment [12]. The interactive map for georeferencing is implemented using the MapBox service provided by the “react-map-gl” suite. An up-to-date version of the tool is available for downloading at Docker Hub (https://hub.docker.com/r/integrativebioinformatics/outbreak) together with the information on how to install and run the software locally.

## Data preparation and input

**Fig. 1:**
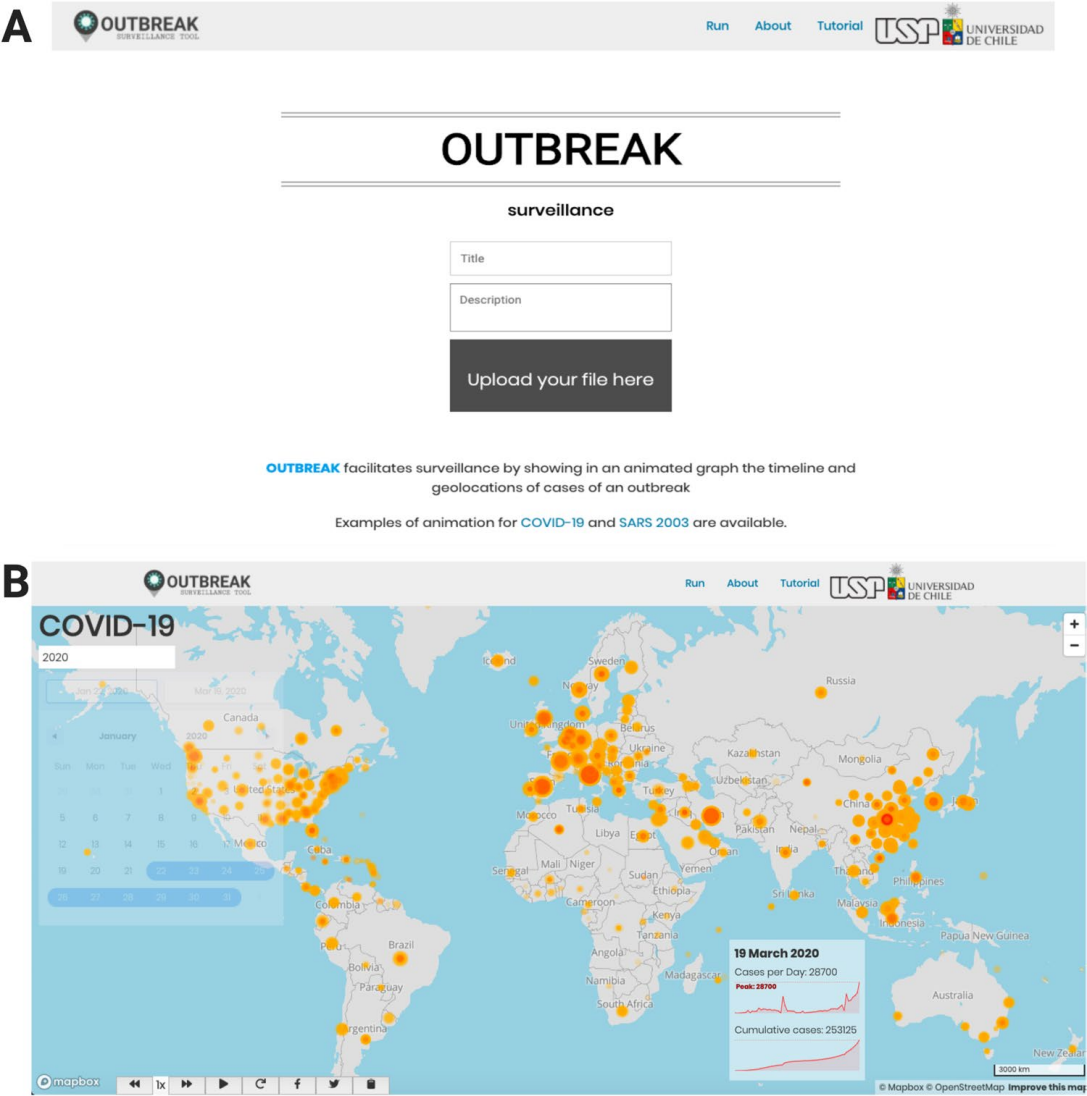
General overview of OUTBREAK Tool. **A** Run section of the tool, where the user uploads the input file and inserts the title and brief description of the surveillance analysis to be performed. **B** Result section showing the dynamic interactive map generated after running the tool

OUTBREAK's input file consists of geographical (latitude and longitude) and temporal (date) information. Users are required to provide this information in a tab-delimited (.txt) file format. The file needs at least four columns with fixed column names as Label, Latitude, Longitude, and Date; representing respectively the row identifier (first column), geographical coordinates (second, third columns) and temporal information (fourth column). The optional columns include the color, size, and number of occurrences for each point (as shown in Table 1). The user can upload the file on the default (Run) page (Fig. 1A), along with a fill-in form with the title and a brief description, later displayed on the interactive map (Fig. 1B). The optional columns help in differentiating the points of interest on the interactive map. For instance, in the example visualization of the COVID-19 pandemic, the reported cases are represented in orange, whereas the associated deaths in red (Fig. 1B).

**Table 1:** General description of the input file for generating interactive maps in OUTBREAK. A description and necessary titles of mandatory and optional columns are displayed

Column title	Data description
Mandatory columns	
Label	ID of respective Latitude and Longitude pairs
Latitude	Latitude value (Decimal Degrees)
Longitude	Longitude value (Decimal Degrees)
Date	Date in day-month-year format
Optional columns	
Color	The names of user-defined colors for representing data on the interactive map. One color to be assigned to each set of data
Size	Size of the points displayed on the interactive map

## Outbreak surveillance and interactive map exploration

**Fig. 2:**
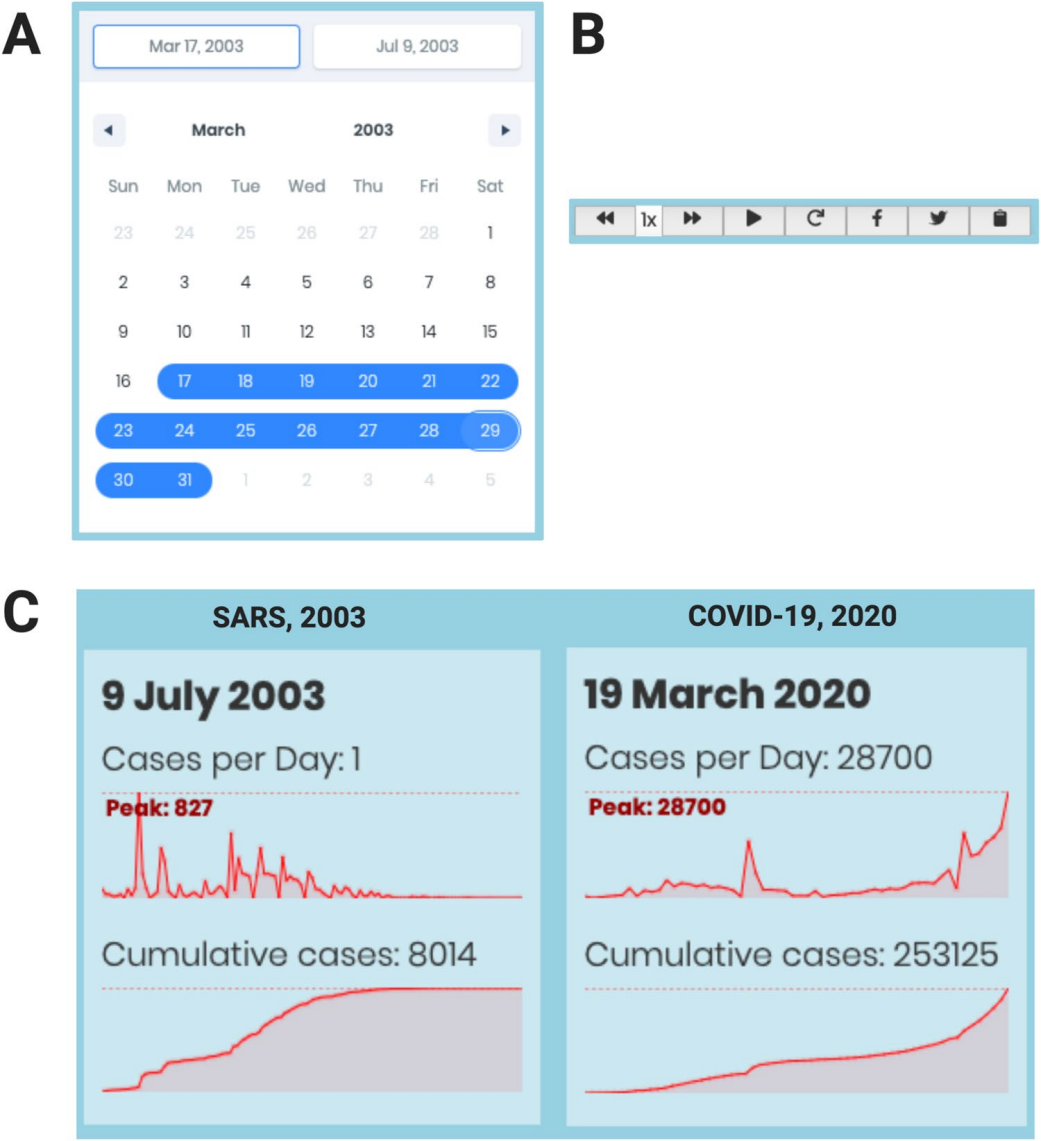
Different features available in OUTBREAK.** A** Calendar input-box to select the period of interest for surveillance. **B** Control menu of the video generated by the OUTBREAK. The user can rewind or fast-forward the animation, with further options to play, pause, restart, or share the video on social media or by copying its URL to the clipboard. **C** Line-graph animation generated while running the tool, showing the cases per day and cumulative cases for SARS 2003 and COVID-19 2020 outbreaks

Upon file submission, users can select a date range on the calendar component to visualize the evolution of an outbreak in a particular period (Fig. 2A). The user can generate animations by hitting the “play” button at the bottom-left of the page (Figs. 1B and 2B). It is possible to change the speed of animation and share it on social media websites. Two dynamic graphs generated on the bottom-right of the page represent the number of cases per day and the cumulative number of cases, respectively. Both graphs dynamically change according to the period previously specified in the calendar. The graph boxes can be dragged and dropped to facilitate inspection of the interactive map. An example demonstration of these features using SARS 2003 and COVID-19 data is shown in Fig. 2C.

**Fig. 3:**
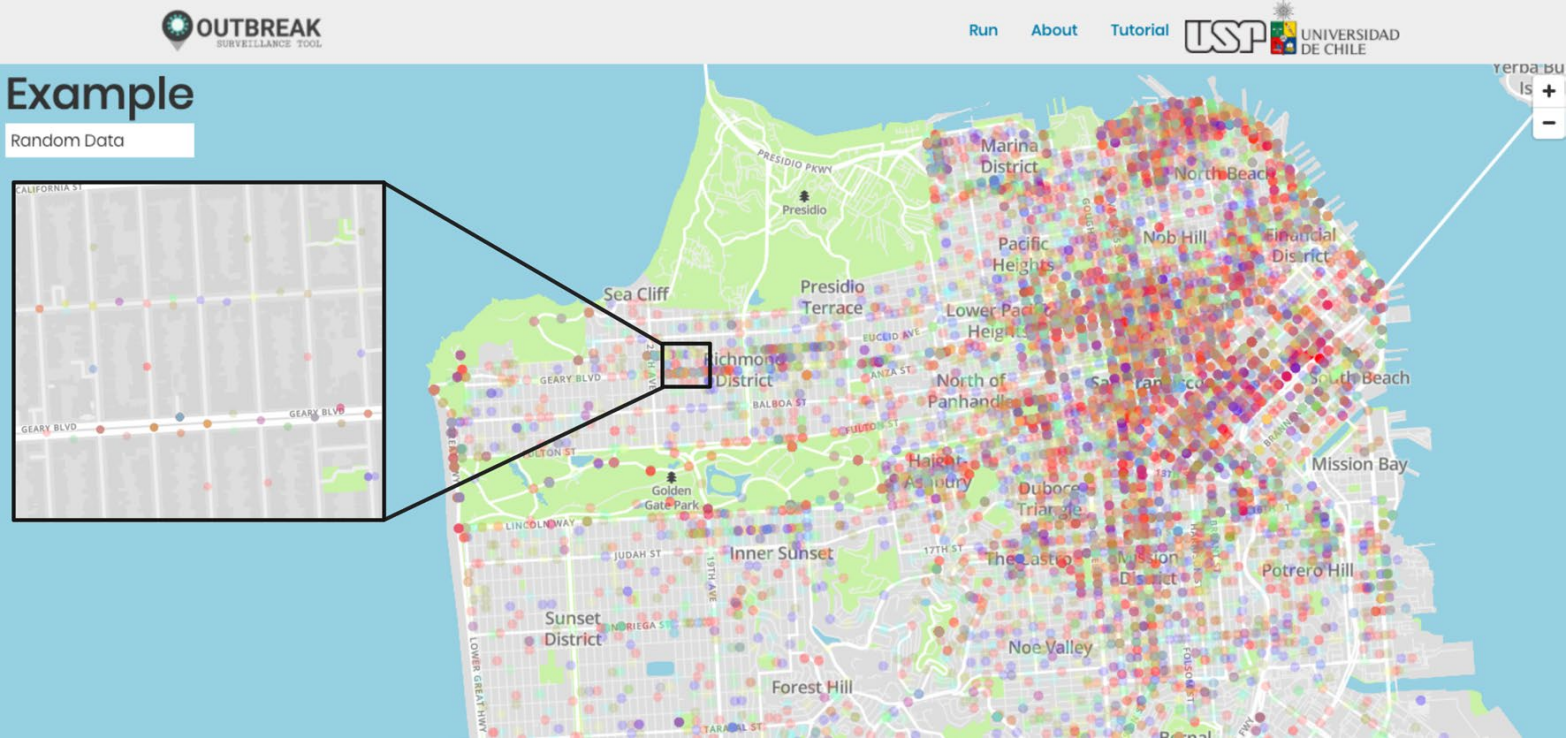
Zoom and color features. Example of surveillance of a hypothetical epidemic in San Francisco using OUTBREAK, using the car incidents dataset from the San Francisco Open Data Portal (https://datasf.org/opendata/). Some key features of the tool are illustrated, such as the use of different colors to show the studied cases, and zoom-feature to investigate particular cases and retrieve the information at a neighborhood, street or even at a single building level

Epidemiologists and decision-makers may need to classify the cases by multiple criteria such as incoming cases, ethnicity, sex, and age, among others, to implement comprehensive disease surveillance. For such classification purposes, OUTBREAK enables the users to apply different colors for each variable of interest (Fig. 3). Finally, the zoom-in feature allows deeper surveillance of an outbreak in the place of interest at different levels, such as neighborhood, street, and even at a single building level (Fig. 3). To demonstrate the above features, we used the car incidents dataset from the San Francisco Open Data Portal (https://datasf.org/opendata/) as a hypothetical epidemic, where each type of incident is shown in a different color (https://outbreak.sysbio.tools/animation/EXAMPLE).

## Conclusion

OUTBREAK is a web-based tool that permits easy surveillance of any epidemiological data. The tool enables the user to monitor epidemic data at various geographical levels – global, country, city, neighborhood, street, or a building. Moreover, the tool allows viewing the change in daily and cumulative case numbers in a graphical format. The input file needs to consist of only geographical coordinates (in decimal degrees format) and dates (dd/mm/yyyy) to generate the graphics. Besides epidemic surveillance, other potential applications include animal migration and population studies. In summary, OUTBREAK aims to enable user-friendly interactive and dynamic maps that can help in the spatial and temporal exploration of an epidemic. We hope this tool will guide the health authorities and decision-makers in making effective interventions to minimize the undesirable impacts of the current and future outbreaks.

## Data Availability

The datasets generated and analysed during the current study are available in the OUTBREAK website at https://outbreak.sysbio.tools/ under MIT license.

## Abbreviation

WHO: World Health Organization
